# 

**Published:** 1885-08

**Authors:** 


### CONTENTS.

ORIGINAL COMMUNICATIONS—

The Lex Coronaria. By Hooper Alexander, Esq., Atlanta, Ga..... 321

Report on Surgery for the Seventh Congressional District. By Everard H. Richardson, M. D., Cedartown, Ga.... 329

A Case of Rattlesnake Bite Successfully Treated with Permanganate of Potash. By B. Hall Smith, M. D.... 338

Phytolacca Decandra in Orchitis. By W. O'Daniel, Bullards, Ga. 340

A Case of Glaucoma the Result of Injudicious Use of Atropia. By J. M. Hull, M. D., Augusta, Ga.... 341

Rupture of Small Intestine and Bladder, From Apparently Inadequate External Violence. By R. P. Huger, M. D., Anniston, Ala...... 344

CLINIC REPORTS—

Glaucoma. By A. W. Calhoun, M. D., Atlanta, Ga.... 348

Post Mortem Examination of a Dirt-Eater. By Drs. Williams and McHatton, Macon, Ga.... 351

BOOK REVIEWS—

Hay Fever and its Successful Treatment. By Chas. E. Sajons, M. D. 354

The Wasting Diseases of Infants and Children. By Eustace Smith, M. D.... 354

Poisons—Their Effects and Detections. By Alexander Wynter Blyth.... 355

Urinary and Renal Derangements and Calculous Disorders. By Lionel S. Beale, M. D.... 356

Perils of American Women. By G. L. Austin.... 357

The Oleates. By John V. Shoemaker, M. D.... 357

Books and Pamphlets Received.... 358

EDITORIAL—

The International Medical Congress.... 360

An Act to Establish a State Board of Medical Examiners and Licensers.... 362

Asiatic Cholera.... 368

Items.... 372

